# The B1 Domain of Streptococcal Protein G Serves as a Multi-Functional Tag for Recombinant Protein Production in Plants

**DOI:** 10.3389/fpls.2022.878677

**Published:** 2022-04-25

**Authors:** Shi-Jian Song, Hai-Ping Diao, Byeongho Moon, Areum Yun, Inhwan Hwang

**Affiliations:** Department of Life Science, Pohang University of Science and Technology, Pohang, South Korea

**Keywords:** plant-based molecular pharming, *Nicotiana benthamiana*, biopharmaceutical proteins, GB1, protein folding

## Abstract

Plants have long been considered a cost-effective platform for recombinant production. A recently recognized additional advantage includes the low risk of contamination of human pathogens, such as viruses and bacterial endotoxins. Indeed, a great advance has been made in developing plants as a “factory” to produce recombinant proteins to use for biopharmaceutical purposes. However, there is still a need to develop new tools for recombinant protein production in plants. In this study, we provide data showing that the B1 domain of Streptococcal protein G (GB1) can be a multi-functional domain of recombinant proteins in plants. N-terminal fusion of the GB1 domain increased the expression level of various target proteins ranging from 1.3- to 3.1-fold at the protein level depending on the target proteins. GB1 fusion led to the stabilization of the fusion proteins. Furthermore, the direct detection of GB1-fusion proteins by the secondary anti-IgG antibody eliminated the use of the primary antibody for western blot analysis. Based on these data, we propose that the small GB1 domain can be used as a versatile tag for recombinant protein production in plants.

## Introduction

Advances in life science have led to the production of recombinant proteins that can be used for various purposes. The first recombinant protein, insulin, was produced for use in humans as a protein drug (Dingermann, 2008). Now, a large number of recombinant proteins, such as antibodies and vaccines, and enzymes are being used as pharmaceuticals (Soler and Houdebine, 2007; Oliveira et al., 2011; Frenzel et al., 2013). Moreover, the area to which these recombinant proteins can be used continues to expand. Thus, the demand to produce more diverse recombinant proteins is increasing. Recombinant proteins can be produced in all kinds of living organisms. Animal cells are largely used for therapeutic proteins (Wurm, 2004; Grillberger et al., 2009). Bacteria and fungi are also convenient systems for recombinant protein production (Specht et al., 2010; Chen, 2012; García-Fruitós, 2012). As a recombinant protein production platform, plants are a more recently developed system (Sainsbury, 2020). These systems have specific advantages and disadvantages.

Compared to animal and bacterial systems, the plant system was introduced most recently (Buyel, 2019; Chung et al., 2021; Schillberg and Finnern, 2021). Thus, the plant system is still in need of improvement in various aspects. In developing plants as a recombinant production platform, the main focus has been to increase the protein production level (Schillberg et al., 2019). Various approaches have been used to increase the expression levels of recombinant proteins. In animal cells, the most powerful approach to increase the expression level is to use drug-induced gene amplification (Hunter et al., 2019). However, the same approach has not been developed in plants. Instead, a similar effect was obtained by using RNA or DNA virus-based vectors that rely on amplification of the target at the level of mRNA or DNA, respectively (Mardanova et al., 2017; Abrahamian et al., 2020). Another powerful approach has been to use the integration of the target gene into plastid chromosomes, leading to a great increase in the level of recombinant proteins in transgenic plants (Adem et al., 2017; Dyo and Purton, 2018). For instance, human somatotropin (hST) recombinant proteins accumulated to the level of more than 7% total soluble protein through the transplastomic transformation approach, which was more than 300-fold higher than the nuclear transgenic approach with a similar gene (Staub et al., 2000). In addition, there have been various approaches to increasing efficiency at the translational level. One approach to increase the expression level in plants was to insert a small domain with multiple *N*-glycosylation sites (Kang et al., 2018). Additionally, various 5’ untranslated sequences were shown to increase the expression level (Kim et al., 2014).

In general, these approaches were successful in increasing protein production levels in plants. However, despite these advances, there is still a big challenge in recombinant protein production, namely the great degree of variation in yield depending on the type of target protein (Hammarström et al., 2006). This is a problem not only in the plant platform but also in other platforms as well (Mancia et al., 2004; Thoring et al., 2017). The causes underlying the variation in protein yield are not fully understood. Yield variation may be due to the intrinsic properties of target genes or target proteins caused at many different levels or by many different mechanisms depending on individual target genes or proteins. One simple approach to address this problem is to optimize codon usage according to the platform. Indeed, the optimization of heterologous genes to the expression host greatly improves the expression level. Another problem may be caused by the folding of the target proteins. The coexpression of chaperons leads to an increase in the protein level (Hammond et al., 1994; Hebert et al., 1995). Furthermore, CRT (calreticulin) of humans leads to an increase in the expression of HIV envelope glycoproteins in plants (Margolin et al., 2020). The stability of recombinant proteins in a foreign environment can cause limitations in the increase in production levels. The fusion of soluble tags to recombinant proteins is a promising strategy that has been used in the production of bioactive proteins (Esposito and Chatterjee, 2006; Hung et al., 2014). The fusion of foreign domains can lead to an increase in production yield. This was thought to have resulted from enhanced folding or stability. These include GST, MBP, SUMO, and GB1 domains, which have been shown to increase protein solubility. In *Escherichia coli*, the GB1 domain of Streptococcal protein G, an antibody binding protein, leads to an increase in the expression level when it is fused to a target protein. GB1, consisting of 56 aa residues, can be divided into two motifs, N- and C-terminal motifs containing 40 and 16 aa residues, respectively. The GB1 domain forms a compact fold that enhances solubility. The increase in the expression level of GB1 fusion proteins was thought to occur *via* the enhancement of protein folding.

In this study, we explored the possibility of using the GB1 domain to enhance protein production in plants. Here, we provide evidence that the fusion of GB1 to the *N*-terminus of various proteins leads to an increase in the production level by enhancing transcription, translation, and stability. Moreover, we showed that the GB1 domain can also serve as an epitope tag that can be detected by western blot analysis using only the secondary anti-IgG antibody.

## Results

### The N-Terminal Fusion of GB1 to GFP Significantly Improves the Expression of GFP in *Nicotiana benthamiana*

**FIGURE 1:**
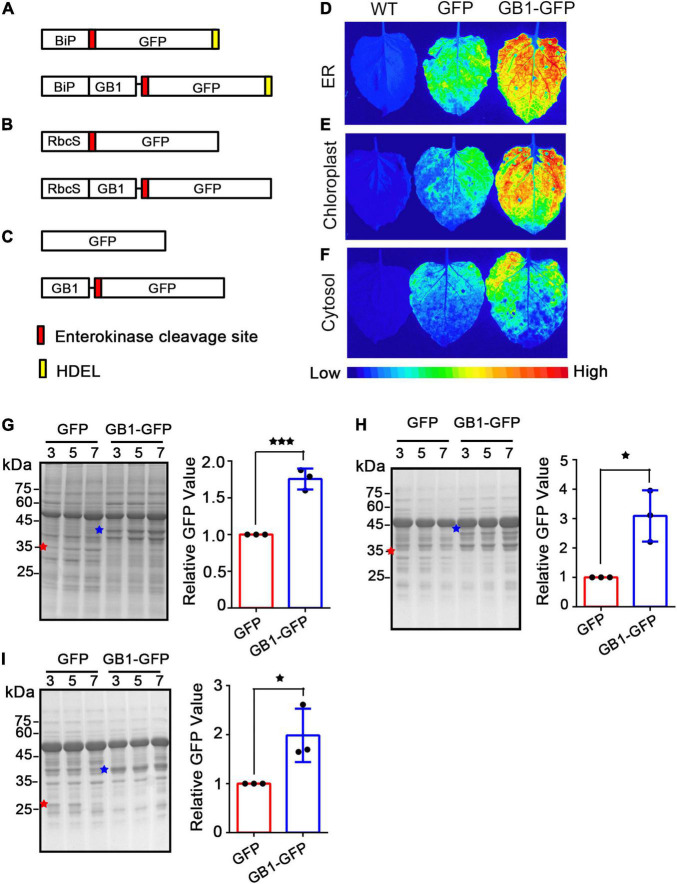
GB1 dramatically increases the expression level of GFP in the leaves of *Nicotiana benthamiana*. **(A–C)** Schematic representation of constructs. *BiP-EK-GFP-HDEL* and *BiP-GB1- EK-GFP-HDEL* **(A)**, *RbcS_*tp*_-EK-GFP-HDEL* and *RbcS_*tp*_-GB1-EK-GFP-HDEL* **(B)**, and *GFP-HDEL* and *GB1-EK-GFP-HDEL* **(C)**. BiP, the leader sequence of BiP; RbcS_*tp*_, transit peptide of the rubisco complex small subunit. **(D–F)** Images of GFP fluorescence. GFP fluorescent signals of the constructs targeted to ER **(D)**, chloroplasts **(E)**, and cytosol **(F)** were measured from infiltrated leaves at 3 days post infiltration (dpi). **(G–I)** Coomassie brilliant blue (CBB)-stained GFP bands. Total protein extracts from infiltrated *N*. *benthamiana* leaves at 3, 5, and 7 dpi were separated by SDS-PAGE and stained with Coomassie brilliant blue. ER **(G)**, chloroplast **(H)**, and cytosol **(I)**-localized GFP (red asterisks) and GB1-GFP (blue asterisks). Band intensity was quantified and represented as a relative value to the GFP alone. Three independent experiments were carried out to quantify the signal intensity. Results in panels **(G,I)** are the mean ± SD (*n* = 3). Asterisks indicate a significant difference (Student’s *t*-test; one asterisk and three asterisks indicate *P* < 0.05 and *P* < 0.001, respectively).

To examine whether the GB1 domain has any beneficial effect on the production of recombinant proteins in plants, we fused it to the N-terminus of a target protein, green fluorescent protein (GFP), as a model protein, thereby yielding *GB1-GFP*. GFP has been widely used as a model protein (Leuzinger et al., 2013; Yamamoto et al., 2018). In plants, the ER and chloroplasts are the two main places for storing recombinant proteins. Thus, the leader sequence of BiP or the transit peptide of RbcS was fused to the N-terminus of GB1-GFP to yield *BiP-GB1-GFP* or *RbcS(tp)-GB1-GFP*, respectively. In addition, we generated two constructs, BiP-GFP and RbcS(tp)-GFP, as controls (Figures 1A,B). These recombinant constructs, together with the cytosolic localized reporter construct *GB1-GFP* and *GFP* alone (Figure 1C), were expressed in *Nicotiana benthamiana via Agrobacterium*-mediated infiltration. The expression of GFP was examined at various time points after infiltration. First, the expression level was examined using the signal intensity of green fluorescence. The signal was captured by LAS3000 under 488 nm excitation. The GB1-fused GFP showed a significantly higher fluorescence signal than GFP without GB1 at 3 days post-infiltration (DPI), regardless of the three subcellular localizations, the ER, chloroplasts, and cytosol (Figures 1D–F). In addition, at five and seven DPI, the GB1-fused GFP gave a higher signal intensity than GFP alone (Supplementary Figures 1A,C,E). These results indicate that the N-terminal fusion of the GB1 domain leads to a higher expression of the fusion protein. To quantify the GB1-mediated increase in the expression level, we calculated the ratio of fluorescence signals between GB1-GFP and GFP at three time points. The ratios for the ER-, chloroplast-, and cytosol-localized proteins were 2.5, 2.5, and 1.7, respectively (Supplementary Figures 1A,C,E), confirming that GB1 fusion leads to dramatic increases in the expression level.

To corroborate this finding, we analyzed the expression levels by western blot analysis using an anti-GFP antibody. Again, the expression level of GB1-GFP was significantly higher than GFP alone in all three locations, the ER, chloroplast, and cytosol (Supplementary Figures 1B,D,F). However, the GB1 domain is derived from protein G, the antibody-binding protein. The GB1 domain alone has the ability to bind to the Fc domain of IgG, indicating that the GB1 domain can be detected by the secondary antibody during western blot analysis. Thus, western blot analysis cannot be used for the quantification of proteins. Hence, instead of western blot analysis employing antibodies, we separated the total protein extracts from *N*. *benthamiana* by SDS-PAGE and stained them with Coomassie brilliant blue (CBB). We were able to detect GFP and GB1-GFP by CBB staining. Furthermore, the levels of GB1-GFP were increased by 1. 7-, 3. 1-, and 2.0-fold compared to the GFP alone in the ER, chloroplasts, and cytosol, respectively (Figures 1G–I), confirming that the GB1 domain leads to an increase in the expression level of fusion proteins.

**FIGURE 2:**
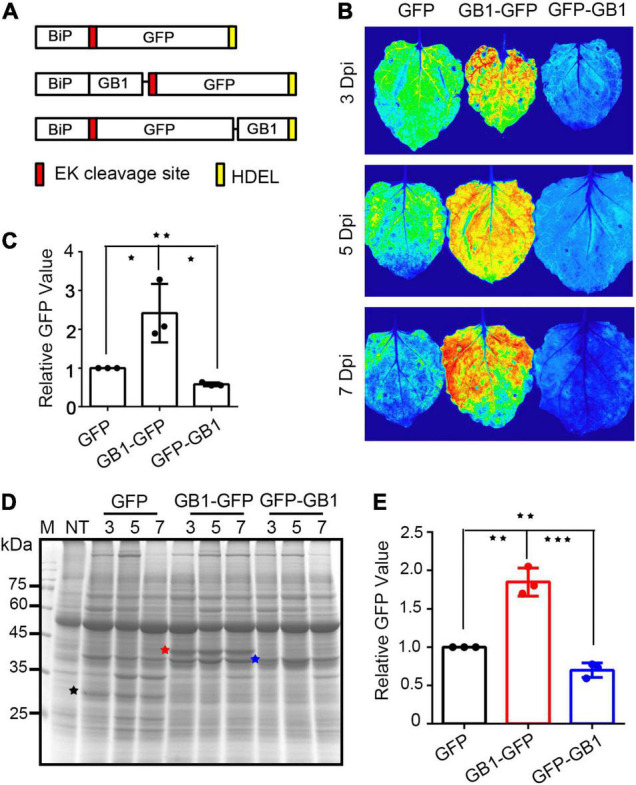
C-terminal fusion of GB1 does not enhance the expression level of GFP in the ER of *Nicotiana benthamiana*. **(A)** Schematic representation of constructs. *BiP-EK-GFP-HDEL* (top), *BiP-GB1-EK-GFP-HDEL* (middle), and *BiP-EK-GFP-GB1-HDEL* (bottom). **(B)** Images of GFP fluorescence. The indicated constructs were expressed in leaf tissues of *N*. *benthamiana via* agroinfiltration, and images were taken at 3, 5, and 7 DPI. GFP (top), GB1-GFP (middle), and GFP-GB1 (bottom). **(C)** Quantification of GFP fluorescent signals. Signal intensity was quantified using three DPI samples. Three independent experiments were carried out. **(D)** GFP level by CBB staining. Total protein extracts from leaves harvested at 3, 5, and 7 DPI were separated by SDS-PAGE and stained with CBB. GFP band intensity was quantified from the CBB-stained gel after SDS-PAGE. **(E)** Quantification of GFP levels. The GFP bands in Figure **(D)** were quantified and represented as relative values. Results for panels **(C,E)** are the mean ± SD (*n* = 3). Asterisks indicate a significant difference (Student’s t-test; one, two, and three asterisks indicate *P* < 0.05, 0.001 < *P* < 0.05, and *P* < 0.001, respectively).

Next, we asked whether the location of the GB1 domain had any effect on the expression of fusion proteins. The GB1 domain was fused to the C-terminus of BiP-GFP to yield BiP-GFP-GB1. Three constructs, BiP-GFP, BiP-GB1-GFP, and BiP-GFP-GB1, were introduced into the leaf tissues of *N*. *benthamiana via Agrobacterium*-mediated infiltration. The expression levels of GFP were examined by fluorescence imaging. In contrast to the N-terminally fused construct BiP-GB1-GFP, the C-terminally fused construct BiP-GFP-GB1 did not show any increase in expression level (Figure 2). In fact, the signal intensity of BiP-GFP-GB1 was even lower than that of BiP-GFP. Together, these results indicate that the location of the GB1 domain in the fusion protein is critical for the effect on the protein expression level.

### N-Terminal GB1 Enhances the Expression of Recombinant Biopharmaceutical Proteins in *Nicotiana benthamiana*

**FIGURE 3:**
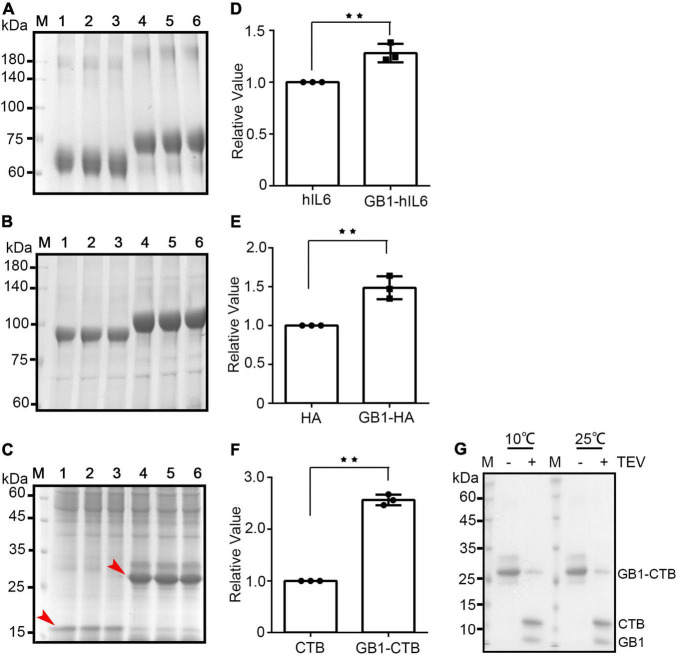
GB1 enhances the expression of various target proteins in *Nicotiana benthamiana*. **(A–C)** SDS-PAGE analysis of target protein levels. The indicated target proteins were transiently expressed in *Nicotiana benthamiana*. These proteins from 0.1 g infiltrated tissues for each sample were purified using Ni^2+^-NTA affinity column chromatography and separated by SDS-PAGE. The gels were stained with CBB. **(A)** Lanes 1–3, purified hIL6; lanes 4–6, GB1-hIL6. **(B)** Lanes 1–3, purified HA; lanes 4–6, GB1-HA. **(C)** Lanes 1–3, purified CTB; lanes 4–6, GB1-CTB. Red arrows indicate the target proteins. **(D–F)** Quantification of signal intensity. The signal intensity of target protein bands in Figures **(A–C)** was quantified and represented in panels **(D–F)**, respectively, as relative values. Results in panels **(D–F)** are the mean ± SD (*n* = 3). Asterisks indicate a significant difference (Student’s *t*-test; two asterisks indicate 0.001 < *P* < 0.05). **(G)** SDS-PAGE (15%) analysis of GB1-CTB cleaved by TEV protease at 10 and 25^°^C overnight.

The effect of the GB1 domain on the expression level of GFP prompted us to examine its effect on various target proteins. GFP is well-known to have good solubility and expression in plants. To further test the functionality of the GB1 domain in increasing the expression levels of recombinant proteins, we selected two proteins, human interleukin 6 (hIL-6) (Islam et al., 2019) and hemagglutinin (HA) of H9N2 (Song et al., 2021). The recombinant constructs, BiP-MP-CBM3-SUMO-hIL6-HDEL (hIL6 in short) and BiP-HA^*H*9*N*2^-mCor1-LysM-His-HDEL (HA^*H*9*N*2^ in short) were tested in *N*. *benthamiana*, and they showed good expression. To test the effect of the GB1 domain on the expression of hIL6 and HA^*H*9*N*2^, the GB1 domain was fused to the BiP leader sequence to yield BiP-GB1-MP-CBM3-SUMO-hIL6-HDEL (GB1-hIL6 in short) and BiP-GB1-HA^*H*9*N*2^-mCor1-LysM-His-HDEL (GB1-HA^*H*9*N*2^ in short), respectively. These constructs, with or without the GB1 domain, were transiently expressed in leaf tissues of *N*. *benthamiana via Agrobacterium*-mediated infiltration. First, the expression of these constructs was examined by western blot analysis using anti-CBM3 and anti-His antibodies for the GB1-hIL6 and GB1-HA^*H*9*N*2^ recombinant proteins, respectively. Both hIL6 and HA^*H*9*N*2^ recombinant proteins with and without the GB1 domain were expressed in *N*. *benthamiana* (Supplementary Figures 2A,B). Those with the GB1 domain showed a much stronger signal intensity. However, the signal intensity of western blot can be biased toward those with the GB1 domain. Thus, to quantify the expression level, we purified both GB1-hIL6 and hIL6 from 0.1 g of infiltrated tissues of each using microcrystalline cellulose (MCC) beads *via* the MCC-binding affinity of the CBM3 domain of the recombinant proteins. The proteins bound to the MCC beads were released by boiling in SDS buffer and separated by SDS-PAGE (Figure 3A). The gels were stained with CBB, and the signal intensity of bands was quantified. The level of GB1-hIL6 was higher by 28% compared to that of hIL6 (Figure 3D). Next, we purified HA^*H*9*N*2^ and GB1-HA^*H*9*N*2^ from infiltrated tissues of each by using Ni^2+^-NTA beads, followed by SDS-PAGE analysis (Figure 3B). The level of GB1-HA^*H*9*N*2^ was higher by 48% compared to that of HA^*H*9*N*2^ (Figure 3E). Both two recombinant proteins have been quantified in the previous studies. The yield of purified hIL6 was approximately 18.5 μg/g FW leaf tissues at near homogeneity (Islam et al., 2019), and the expression level of trimeric HA^*H*9*N*2^ was 150 μg/g FW leaf tissues (Song et al., 2021). We estimated that the expression level of GB1-hIL6 and GB1-HA^*H*9*N*2^ is approximately 23.7 μg/g FW and 222 μg/g fresh weight, respectively, according to the relative ratio with hIL6 and HA^*H*9*N*2^.

To further expand our findings, we tested the effect of the GB1 domain on the expression of the cholera toxin B subunit (CTB), a natural homopentamer protein, without any other translational enhancing domains. When CTB as recombinant construct *BiP-CTB-His-HDEL* (CTB in short) was expressed in *N*. *benthamiana*, it was expressed at low levels. The GB1 domain was fused next to BiP to yield BiP-GB1-CTB-His-HDEL (GB1-CTB in short) (Supplementary Figure 2C). These two constructs, *CTB* and *GB1-CTB*, were transiently expressed in *N*. *benthamiana via Agrobacterium*-mediated infiltration. First, their expression was examined by western blot using an anti-His antibody, confirming their expression (Supplementary Figure 2F). Next, to quantify the expression levels of these two recombinant proteins, both CTB and GB1-CTB were purified from 0.1 g infiltrated tissues using Ni^2+^-NTA affinity column chromatography. The purified proteins were separated by SDS-PAGE, and the gel was stained with CBB (Figure 3C). The GB1 domain led to an increase of CTB recombinant protein level by 2.6-fold (Figure 3F). The GB1-mediated increase in expression was higher with CTB than with other target proteins. Together, these results showed that the N-terminal GB1 broadly enhanced the expression of recombinant proteins in plants. GB1 is a domain that increased the expression level of recombinant proteins in plants and also can be used as an epitope tag for detection during western blot analysis. However, in a certain case, it is desirable to remove the GB1 tag. We tested the possibility of removal of the GB1 domain from GB1-containing fusion proteins, GB1-CTB that had a tobacco etch virus (TEV) site in between GB1-CTB. GB1-CTB was purified using Ni^2+^-NTA affinity column chromatography and treated with TEV protease at 10 or 25^*o*^C overnight. Most of GB1-CTB recombinant protein was successfully cleaved by TEV at both conditions (Figure 3G). CTB released from GB1-CTB by TEV was slightly smaller in size than BiP-CTB-His, likely due to BiP leader sequence.

### The GB1 Domain Enhances Both Transcriptional and Translational Efficiency

**FIGURE 4:**
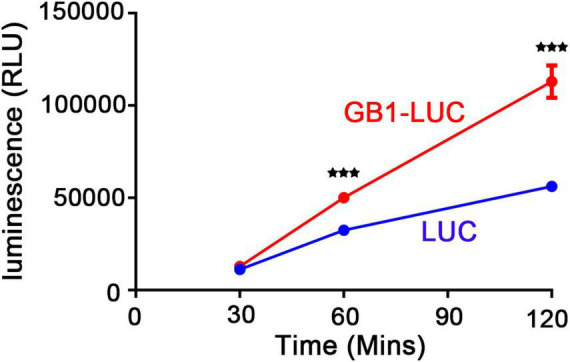
GB1 fusion leads to an enhanced translation rate *in vitro*. LUC and GB1-LUC were transcribed and translated *in vitro* in wheat germ extracts. During the reaction, 5 μl were taken from the 50 μl reaction volume at 30, 60, and 120 min and diluted 24-fold. The RLUC activity was measured using the Renilla Luciferase Assay System. Three independent translation reactions were carried out. The error bar is the mean ± SD (*n* = 3). Asterisks indicate a significant difference (Student’s *t*-test; three asterisks, *P* < 0.001).

The previous study suggested that GB1 is a soluble-promoting tag that can enhance the solubility of target protein for better folding and, in turn, enhance the final yield in an *E*. *coli* expression system (Zheng et al., 2016). To understand the mechanism by which the GB1 domain led to high expression of fusion proteins in *N*. *benthamiana*, we first examined the effect of GB1 on translational efficiency. We generated LUC and GB1-LUC constructs, and the translation rate was examined in wheat germ extracts *in vitro* by measuring the bioluminescence. The signal intensity of luminescence was almost the same at the 30 min time point. However, luminescence signals of GB1-LUC were increased by 1.6- and 2.0-fold to that of LUC alone at 60 and 120 min time points, respectively (Figure 4), indicating that GB1 enhances translation of the fusion protein.

Next, we examined whether the GB1 domain had any effect on transcriptional efficiency. We performed qRT-PCR analysis of the target genes. GFP and GB1-GFP were transiently expressed in *N*. *benthamiana* leaves *via Agrobacterium*-mediated infiltration. Total RNA was used for qRT-PCR analysis. The transcript level of GB1-GFP was increased by 1.7-fold compared to that of GFP alone (Supplementary Figure 3A). To corroborate this finding, we tested the effect of GB1 on another target, CTB. The transcript levels of GB1-CTB and CTB were examined by qRT-PCR (Supplementary Figure 3B). Again, the transcript level of GB1-CTB was higher than that of CTB, although the increment was smaller compared to that of GFP. These results suggest that GB1 can also increase the transcription efficiency of fusion genes.

### The Hydrophobic Cluster of GB1 Enhances the Thermal Stability of Recombinant Cholera Toxin B Subunit but Does Not Affect the High Expression

**FIGURE 5:**
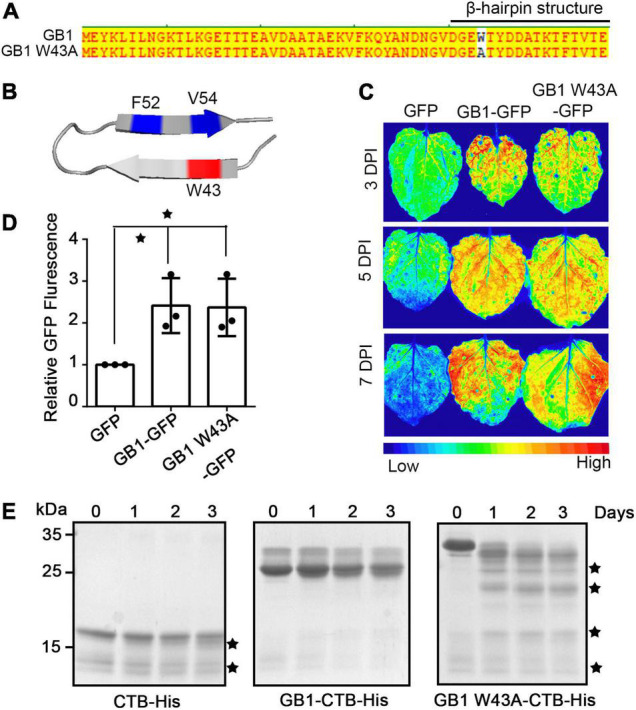
The β-hairpin structure of GB1 is critical for the thermal stability of CTB but has no contribution to the increase in protein expression. **(A)** Protein sequences of GB1 and GB1[W43A]. **(B)** The β-hairpin structure of GB1. **(C)** Images of GFP fluorescence. The indicated constructs were expressed in *Nicotiana benthamiana* leaves, and images were taken at three DPI. **(D)** Quantification of GFP signals. GFP signals in Figure **(C)** were quantified and represented as relative values. The error bar is the mean ± SD (*n* = 3). Single Asterisks indicate a significant difference (Student’s *t*-test, *P* < 0.05). **(E)** Thermal stability of GB1 and GB1[W43A]-fused CTB. The indicated constructs were expressed in *N*. *benthamiana via Agrobacterium*-mediated infiltration. Proteins were purified using Ni^2+^-NTA affinity column chromatography. Purified proteins (2 μg) were incubated at 60^*o*^C for 1, 2, and 3 days and separated by SDS-PAGE. The gel was stained with CBB. Black asterisks indicate protein breakdown products.

Recombinant proteins that have been used as biopharmaceuticals are generally thermal stable (Huus et al., 2005; Wakankar et al., 2010; Mulinacci et al., 2011). However, certain recombinant proteins that are under development as protein drugs have a problem of poor stability (Willuda et al., 1999; Arndt et al., 2003). The 16-residue hairpin of GB1 exhibits many basic features involved in protein folding, including stabilization by both hydrogen bonding and hydrophobic interactions (Muñoz et al., 1997). In GB1, W43 interacts with F52 and V54, forming a hydrophobic cluster that has been proven to play a key role in the stabilization of the GB1 structure, which, in turn, contributes to stabilizing the GB1 fusion protein (Figure 5B; Muñoz et al., 1997). We examined whether the stabilization effect of the GB1 β-hairpin on GB1 fusion proteins contributes to the high expression of the GB1 fusion protein. We introduced the W43A (Figure 5A) point mutation to GB1 to abolish the hydrophobic bond of GB1. GB1[W43A] was fused to GFP to give GB1[W43A]-GFP, and the resulting construct together with *GB1-GFP* was transiently expressed in *N*. *benthamiana*. The expression level was examined by GFP fluorescence in the leaves. The expression levels of GB1-GFP and GB1[W43A]-GFP were similar to each other (Figures 5C,D), indicating that the W43A mutation does not affect the enhancement of protein expression. Next, we examined that whether the β-hairpin structure can enhance the stability of GB1-fused recombinant proteins. Here, we fused GB1[W43A] to cholera toxin B subunit (CTB) and tested its effect on thermal stability. GB1-CTB and GB1[W43A]-CTB were purified using Ni^2+^-NTA affinity column chromatography and incubated at 60^*o*^C for 3 days. Proteins were analyzed by SDS-PAGE. CTB alone showed a gradual increase in protein degradation over time, whereas GB1-CTB was intact even during the 3 days of incubation. In contrast, GB1[W43A]-CTB showed clear degradation even 1 day after incubation (Figure 5E), indicating that W43 plays a role in the stability of the GB1 fusion protein. These results strongly suggest that the hydrophobic cluster of GB1 confers the thermal stability to the GB1-fused recombinant CTB but does not affect the high expression. However, it is not clear whether the thermal stability conferred by GB1 is specific to CTB or is a general phenomenon to other proteins.

### GB1 Can Be Detected by Various Secondary Antibodies Due to Its Affinity for the Fc Domain

**FIGURE 6:**
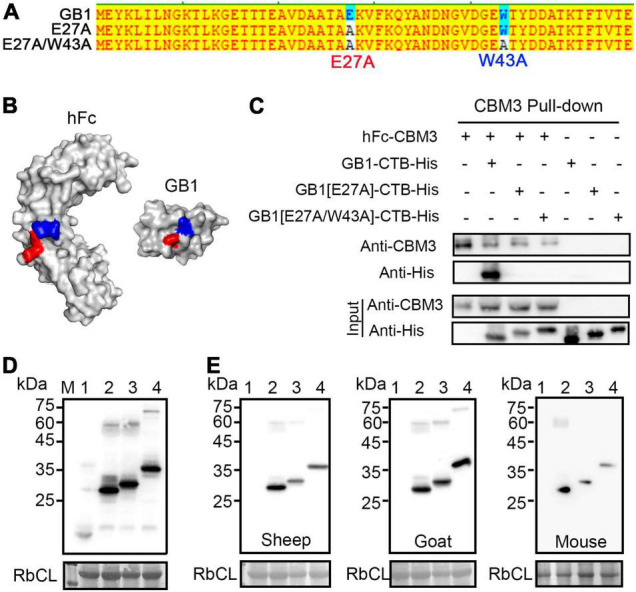
GB1 is directly detected by IgG from various animals. **(A)** Protein sequences of GB1, GB1[E27A], and GB1[E27A/W43A]. Asterisks indicate mutations. **(B)** The 3-D structures of human Fc and GB1. These structures were obtained from the public database. Human Fc, PBD: 1aj7; GB1, PBD: 2gi9. The red color at the GB1 structure indicates E27, which functions as a knob for the hole of Fc (H433 and N434, red); the blue color at the GB1 structure indicates W43, which functions as a hole for the knob of Fc (I253 and S254, blue). **(C)** Strong binding of hFc to GB1 but not GB1 mutants. The indicated constructs were expressed in *Nicotiana benthamiana*, and total protein extracts were subjected to MCC beads-based protein pull-down. The proteins bound to the MCC beads were analyzed by western blotting using anti-CBM3 and anti-His antibodies. **(D)** Western blot analysis. Total protein extracts from *N*. *benthamiana* expressing the indicated constructs were analyzed by western blotting using the anti-His antibody. RbcL stained with CBB was used as a loading control. **(E)** Binding of various IgGs to GB1. Protein extracts used in Figure **(D)** were separated by SDS-PAGE and analyzed by western blotting using sheep anti-Mouse IgG (Sheep), goat anti-Human IgG (Goat), and mouse anti-Goat IgG (Mouse) without primary anti-His antibody. CTB, lane 1; GB1-CTB, lane 2; GB1[E27A]-CTB, lane 3; GB1[W43A]-CTB, lane 4. All constructs contain a His-tag at the C-terminus but are omitted to simplify the description.

Protein G shows the binding affinity to various types of IgG derived from most of the organisms with different binding strengths (Björck and Kronvall, 1984) by its affinity for the Fc, Fab, scFv, and Dab domains (Akerström and Björck, 1986; Choe et al., 2016). Thus, it is possible that the GB1 domain can also bind to IgG from various animals. Indeed, the GB1 binding site on the Fc fragment of human IgG has been dissected in a previous study (Figure 6B; Sloan and Hellinga, 1999). Thus, we first examined the interaction between GB1 and Fc by protein pull-down experiments. We generated a fusion construct, hFc-CBM3, and used it for the pull-down experiments. *hFc-CBD* was co-expressed with *GB1-CTB-His*, *GB1[E27A]-CTB-His*, or *GB1[E27A/W43A]-CTB-His* (Figure 6A) in *N*. *benthamiana via Agrobacterium*-mediated infiltration. Total protein extracts were prepared and used for protein pull-down experiments with microcrystalline cellulose (MCC) beads. The proteins bound to MCC beads were analyzed by western blotting using both anti-CBM3 and anti-His antibodies. GB1-CTB-His, but not GB1[E27A]-CTB-His and GB1[E27A/W43A]-CTB-His, was detected in the MCC beads-bound proteins by the anti-His antibody (Figure 6C). However, after long-term exposure, GB1[W43A]-CTB-His was also detected (Supplementary Figure 5). The results suggest that both mutations E27A and W43A affect the binding to hFc, and the E27A was more detrimental to the binding affinity than the W43A mutation.

The interaction between GB1 and hFc raises the possibility that GB1 can be used as a liner epitope that could be directly detected by associated paratopes of secondary antibodies in western blot analysis. To test this idea, we performed western blot analysis of CTB, GB1-CTB, GB1[E27A]-CTB, and GB1[W43A]-CTB. These recombinant proteins were tagged with the small epitope His (His6). Thus, these proteins were analyzed by western blotting using anti-His antibody (1,000×), followed by anti-mouse IgG as a secondary antibody, or secondary IgG alone from various sources, namely mouse, goat, and sheep without the anti-His antibody, as the primary antibody. As in the case of inclusion of the anti-His antibody as the primary antibody, when we used various secondary IgGs alone, they all detected these GB1 fusion proteins (Figures 6D,E). Wild-type GB1 showed a stronger signal, indicating that the mutations affected the binding affinity to IgG. Both GB1[E27A]-CTB and GB1[W43A]-CTB showed an increased size in SDS-PAGE compared to GB1-CTB. A similar effect was observed when they were fused to GFP, indicating that slower migration is caused by the structural changes caused by the mutations (Supplementary Figure 4). These results confirm that the GB1 domain can be used for western blot analysis as an epitope tag that can be detected using only the secondary IgG of various animals.

## Discussion

In this study, we provide evidence that the fusion of a small domain GB1 leads to significant enhancement of protein yield in plants and gains high stability to the recombinant proteins. The fusion of tags to increase the solubility of aggregation-prone proteins has been widely used in the production of recombinant proteins in *E*. *coli* (Bao et al., 2006; Peti and Page, 2007; Sugase et al., 2008). One of them is the B1 domain of streptococcal protein G (56 aa), which forms a compact fold with high solubility, which can contribute to enhanced solubility to the GB1 fused target protein in *E*. *coli* (Bauer et al., 2009; Hung et al., 2014). GB1 contains two separable domains: N-terminal (1–40 aa) and C-terminal (41–56 aa) domains. The C-terminal domain tends to form a β-hairpin structure that makes it one of the smallest known peptides to fold into a defined structure (Muñoz et al., 1997; Du et al., 2004). The β-hairpin structure has been studied and contributes to much of the basic physics of protein folding, including stabilization by hydrogen bonding and hydrophobic interactions.

In this study, we explored the possibility of the GB1 domain as a tag for the purpose of increasing the protein production yield in plants. We reasoned that the mechanism by which GB1 leads to the high-level production of GB1 fusion proteins recombinant proteins is likely attributed to better folding *via* its solubility-enhancing activity. Various GB1 fusion constructs, when expressed in *N*. *benthamiana*, showed an increase in the level of GB1-fused target proteins with a certain degree of variation compared to untagged versions. This result suggests that the GB1 domain can be used in many different proteins for high-level production in plants. However, the effect of GB1 on the increase in the protein level was strictly dependent on its N-terminal localization. The reason for localization dependency is not clearly understood. One possible explanation is that the N-terminal localized GB1 can contribute to the folding of newly translated proteins. In contrast, *C*-terminal tagged GB1 may not have much chance of contributing to the fold. Indeed, this finding is consistent with the notion of the effect of GB1 on fusion proteins in *E*. *coli* (Bao et al., 2006; Peti and Page, 2007; Sugase et al., 2008). In addition, the effect of the GB1 domain on an increase in the expression level did not show any dependency in the subcellular localization of proteins, indicating that the GB1 domain can be used in many different types of proteins without any restriction on the localization.

We examined the mechanism by which GB1 enhances protein production levels in plants. As mentioned above, it is possible that GB1-mediated folding enhancement is the mechanism underlying high-level protein production in plants as well. However, unexpectedly, the GB1 domain also contributed to the increase in the transcript levels of GB1-fused target genes. Currently, this is not fully understood, since it is part of the coding region but not the promoter or terminator of fusion genes. However, often the nucleotide sequence of the coding region also contributes to the level of transcripts by affecting the transcription efficiency or stability of mRNA. Currently, we have not further addressed these points in this study. Thus, the mechanism underlying the GB1-mediated increase in the level of mRNA is still not fully understood. Another mechanism we examined was translation efficiency *in vitro*. Indeed, GB1-fused LUC was translated at a much higher level compared to LUC alone *in vitro*. Again, this can be attributed to the enhanced folding of GB1-fused LUC compared to LUC alone. However, we cannot rule out the possibility of a higher translation rate of *GB1-LUC* mRNA than of *LUC* mRNA. Together, these results suggest that GB1 positively contributes to the expression of GB1-fused genes at both the transcriptional and translational levels.

Protein G is widely used as a purification resin of antibodies, owing to its ability to bind to the Fc and Fab regions. We found that the GB1 domain alone could bind to hFc. We explored the possibility of using the GB1 domain as an epitope tag for western blot analysis. Many small epitopes, such as His, FLAG, and HA, have been used as epitope tags for western blot analysis. However, these tags require the use of a primary antibody that can specifically detect tags. Subsequently, the primary antibody was detected by the secondary anti-IgG antibody fused to horseradish peroxidase. Most of these primary antibodies are expensive. Additionally, using both primary and secondary antibodies requires a fairly long period of experimental time. GB1-fused CTB was successfully detected by anti-IgG antibodies from various animals without using any primary antibodies. Even though GB1 can be used as a multi-functional tag in the study of molecular farming, its removal should be concerned in the final application of proteins for use as biopharmaceuticals, similar to other tags. The tags are usually removed using specific proteases. The released target proteins without the tag should be further purified, which results in increase in production cost and yield loss. Approaches to reduce cost has been proposed that include the self-cleaving intein tag (Coolbaugh et al., 2017) and a sequence-specific chemical protein cleavage tag (Dang et al., 2019).

In summary, we showed that the small GB1 domain can be a versatile tag for recombinant protein production in plants. First, GB1-fused proteins can be highly expressed and well-folded. Second, GB1-fused recombinant proteins can be detected by the secondary antibody in a cost-effective and time-saving manner. Finally, the GB1 tag can potentially be used for Fc resin-mediated purification, which can be developed in the future.

## Materials and Methods

### Construction of Recombinant Genes

DNA fragments encoding GFP together with an N-terminal enterokinase site (EK) were N-terminally fused with the ER leader peptide of Arabidopsis BiP1 or the transit peptide of Arabidopsis RbcS (RbcS_*tp*_) to yield BiP-EK-GFP-HDEL or RbcS_*tp*_-EK-GFP. The B1 domain (amino acid positions from 1st to 56th) of *Streptococcal* protein G (GB1) was inserted after the ER leader sequence or RbcS_*tp*_ of BiP-EK-GFP or RbcS_*tp*_-EK-GFP to yield BiP-GB1-EK-GFP-HDEL, or C-terminally fused to BiP-EK-GFP to yield BiP-EK-GFP-GB1. The DNA fragments encoding hIL6 and HA^*H*9*N*2^ were prepared from recombinant constructs *BiP-MP-CBM3-SUMO-hIL6-HDEL* and *BiP-HA*^*H*9*N*2^-*mCor1-LysM-His-HDEL*, respectively (Islam et al., 2019; Song et al., 2021), by digesting with *Bam*HI and *Xho*I, and ligated into BiP-GB1-EK-GFP-HDEL digested with *Bam*HI and *Xho*I to yield *BiP-GB1- MP-CBM3-SUMO-hIL6-HDEL* and *BiP-GB1-HA*^*H*9*N*2^-*mCor1-LysM-His-HDEL*, respectively. *CTB* with *Bam*HI and *Xho*I restriction sites at N- and C-terminal ends, respectively, was chemically synthesized (Gene Universal, Inc., Newark, United States). All the constructs were placed under the MacT promoter (Song et al., 2021) and accompanied by RD29B Terminator of Arabidopsis *RD29b*. Primers used in this study are shown in Supplementary Table 1.

### Production of Transient Transgenic Plants

Expression vectors were introduced into *Agrobacterium* strain GV3101 by electroporation. A single colony of *Agrobacterium* harboring expression vectors was inoculated to LB Broth (LPS Solution, Cat. LBL-05) and cultured in an incubator at 28^°^C overnight. Four–five week-old *N. benthamiana* plants grown in a greenhouse at 25^*o*^C with a 16 h light/8 h dark cycle were used for Agroinfiltration by syringe. The infiltrated leaves were harvested at 3, 5, and 7 days post infiltration (DPI) to examine the expression level.

### SDS-PAGE and Western Blot Analysis

Infiltrated leaves were ground and homogenized in protein extraction buffer [PBS buffer containing 1 mM EDTA, 0.5% Triton X-100(v/v), 1 X protease inhibitor cocktail]. The total protein extracts or purified proteins were separated by 7.5–12% SDS-PAGE. Western blot analysis was performed using the mouse anti-His antibody (1: 1,000 dilution, Novus, AD1.1.10), mouse anti-GFP antibody (1: 1,000 dilution, Clontech, Cat. number: 632381), and mouse anti-HA antibody (1: 1,000 dilution, Sigma, H3663). The secondary antibodies used in this study were goat anti-Human IgG conjugated HRP, sheep anti-Mouse IgG conjugated HRP (1: 5,000 dilution, Bethyl Laboratories), and mouse anti-Goat IgG conjugated HRP (1: 5,000 dilution, Santa Cruz Biotechnology, Inc.). Immunoblots were developed with the enhanced chemiluminescence kit (Amersham Pharmacia Biotech, Piscataway, NJ, United States), and images were captured using the LAS3000 system (Fujifilm, Tokyo, Japan).

### Green Fluorescent Protein Fluorescence Acquisition and Quantification

Infiltrated leaves expressing *GFP* or *GB1-GFP* were harvested at 3, 5, and 7 DPI. The GFP fluorescence image of leaves was captured using the LAS3000 system (Fujifilm, Tokyo, Japan). The density of GFP fluorescence from the whole infiltrated leaves was measured by the official software of LAS3000 system (Fujifilm, Tokyo, Japan).

### Ni^2+^-NTA and Microcrystalline Cellulose Beads-Based Affinity Purification

Total protein extracts were prepared from 0.1 g leaf tissues infiltrated with *Agrobacterium* harboring *HA*^*H*9*N*2^, *GB1-HA*^*H*9*N*2^, *CTB*, or *GB1-CTB* using extraction buffer (50 mM Tris-HCl, pH 7.5, 150 mM NaCl, 1 mM DTT, 0.1% [v/v] Triton X-100, 10 mM imidazole, and protease inhibitor cocktail), and incubated with Ni^2+^-NTA agarose beads (Qiagen, Valenica, CA, United States) on a shaker in a cold room for 30 min. Ni^2+^-NTA agarose beads with bound proteins were washed using extraction buffer supplemented with 20 mM imidazole. Target proteins were eluted with 400 mM imidazole in the extract buffer. For MCC bead-based protein purification, total extracts were prepared from 0.1 g of leaf tissues expressing *hIL6* or *GB1-hIL6* using extraction buffer (50 mM Tris-HCl, pH 7.5, 150 mM NaCl, 1 mM DTT, 0.1% [v/v] Triton X-100, and protease inhibitor cocktail) and incubated with MCC beads (Sigma-Aldrich, St. Louis, MO, United States, CAS Number 9004-34-6) on a shaker for 30 min. MCC beads with bound proteins were washed with extraction buffer and boiled in the extract buffer for 10 min to release proteins from MCC beads.

### Coomassie Brilliant Blue Staining and Statistics Analysis

The protein bands separated by SDS-PAGE were stained in Coomassie brilliant blue dyes, Coomassie brilliant blue 0.025% (m/v), methanol 50% (v/v), acetic acid 10% (v/v), ddH_2_O 30%(v/v). The bands in the PAGE were imaging captured by LAS3000 system (Fujifilm, Tokyo, Japan) after de-staining. The bands’ density was measured by using its official software. All the values of western band density or GFP fluorescence density were analyzed using the Student t-test or using the software, GraphPad Prism 6.02. *P*-values ≤ 0.05 were considered statistically significant.

### TEV Cleavage

GB1-CTB proteins (2 μg) purified from the plant extracts were mixed with 0.2 U AcTEV™ Protease (Invitrogen, Cat. 2575015) and 2 μL 20 x TEV buffer, by adding ddH_2_O up to 40 μL. The mixtures with or without TEV protease were incubated at 10 or 25^°^C overnight, followed by SDS-PAGE analysis.

### RNA Extraction and qRT-PCR

Leaf tissues were collected at 3 DPI and ground using stainless steel beads that had been precooled by liquid nitrogen. The total RNA was purified by using GeneJET plant RNA purification kit (Thermo Scientific, Waltham, MA, United States), following the protocol provided by the manufacturer. The final RNA concentration was measured by NanoDrop™ 2000/2000c Spectrophotometer (Thermo Scientific, Waltham, MA, United States). Total RNA (2 μg) was reverse-transcribed to cDNA by MutiScribe Reverse Transcriptase (Thermo Fisher Scientific, REF 4368813) for qRT-PCR. The cDNA (50 ng), primers, and SYBR Green mix (Thermo Fisher Scientific, REF A25742) were mixed for qRT-PCR under the condition of 15 s denaturation at 95^°^C and 20 s annealing at 60^°^C and 30 s extension at 70^°^C with 40 cycles. The primers used in qRT-PCR were shown in Supplementary Table 1.

### Protein Pull-Down Experiments Microcrystalline Cellulose Beads

*hFC-CBM3* was infiltrated into the *N. benthamiana* leaf tissues together with *GB1-GFP-His*, GB1[E27A]-CTB-His, GB1[E27A/W43A]-CTB-His, or GB1[W43A]-CTB-His. Total protein extracts from leaf tissues of these infiltrated plants were incubated with MCC beads followed by washing three times with TBS buffer. The proteins pulled down by MCC beads were released by boiling and analyzed by western blotting using anti-CBM3 or anti-His antibodies.

### *In vitro* Translation and Transcription

Both *BiP-LUC* and *BiP-GB1-LUC* were ligated into the pCS2++(modified from pCS2+) vector digested with *Xba*I and *Pst*I restriction endonucleases. DNA templates in the pCS2++vector were linearized by PCR using two primers covering the SP6 promoter and terminator. Capped mRNA was transcribed in the presence of a cap analog m7G[5′]ppp[5′]G using the mMESSAGE mMACHINE TM SP6 kit (Invitrogen, Cat. AM1340). The *in vitro* transcription was carried out at 37°C for 2 h in the mixture containing 5 mM ATP, 5 mM CTP, 5 mM UTP, 1 mM GTP, 4 mM cap analog m7G[5′]ppp[5′]G. The *in vitro* translation reaction mixture of wheat germ extracts (Promega, Cat. L4130) contained 10 mM creatine phosphate, 50 μg/ml creatine phosphokinase, 5 mM DTT, 2.1 mM magnesium acetate, 53 mM potassium acetate, 0.5 mM spermidine, 1.2 mM ATP, 0.1 mM GTP, 40 μM methionine, 40 μM leucine, 80 μM other amino acids and 40 units RNasin Ribonuclease Inhibitor. *In vitro* synthesized mRNA (8 fmol/μl) was quantified by a NanoDrop™ 2000/2000c Spectrophotometer (Thermo Scientific, Waltham, MA, United States) and added to the reaction mixture and the translation reaction was performed at 25°C for 2 h in a 50 μl total reaction volume. 5 μl from the 50 μl reaction volume were collected at 30, 60, and 120 min points, diluted to 24-fold, and frozen using liquid nitrogen. RLUC activity of samples was measured using the Renilla Luciferase Assay System kit (Promega, E2710). Primers used in this study are shown in Supplementary Table 1.

## Data Availability Statement

The datasets presented in this study can be found in online repositories. The names of the repository/repositories and accession number(s) can be found in the article/Supplementary Material.

## Author Contributions

IH and S-JS contributed to the conception of the study and wrote the manuscript. S-JS and H-PD made the constructs and contributed significantly to analysis and experiments. BM and AY contributed to the *in vitro* translation and vector construction, respectively. All authors contributed to the article and approved the submitted version.

## Conflict of Interest

The authors declare that the research was conducted in the absence of any commercial or financial relationships that could be construed as a potential conflict of interest.

## Publisher’s Note

All claims expressed in this article are solely those of the authors and do not necessarily represent those of their affiliated organizations, or those of the publisher, the editors and the reviewers. Any product that may be evaluated in this article, or claim that may be made by its manufacturer, is not guaranteed or endorsed by the publisher.

## Supplementary Material

Supplementary Figure 1 GB1 dramatically increases the expression of soluble GFP in leaf tissues of *Nicotiana benthamiana*. **(A,C,E)** Images of GFP fluorescence for the ER **(A)**, chloroplast **(C)**, and cytosol **(E)**-localized GFP constructs. Images were taken at 5 and 7 dpi. The GFP signals were quantified at three DPI from three different samples. **(B,D,F)** Western blot analysis. Total protein extracts from leaf tissues harvested at 3, 5, and 7 DPI were analyzed by western blot analysis using the anti-GFP antibody. ER **(B)**, chloroplast **(D)**, and cytosol **(F)**-localized GFP and GB1-GFP at three time points. Lanes 1, 3, and 5 indicate GFP at 3, 5, and 7 DPI, respectively. RbcL stained with CBB was used as a loading control. Lanes 2, 4, and 6 indicate GB1-GFP at 3, 5, and 7 DPI, respectively. Results in panels **(A,C,E)** mean ± SD (*n* = 3). Asterisks indicate a significant difference (Student’s *t*-test; two asterisks, 0.001 < *P* < 0.05).

Supplementary Figure 2 Western blot analysis of the expression of various target genes in *Nicotiana benthamiana*. **(A–C)** Schematic representation of constructs. **(D–F)** Western blot analysis for the expression of various recombinant proteins. *Nicotiana benthamiana* leaf tissues were infiltrated with Agrobacterium harboring MCS-hIL6-HDEL(hIL6) or GB1- MCS-hIL6-HDEL(GB1-hIL6) **(D)**, HA^*H*9*N*2^-mCor1-LysM-His-HDEL or GB1- HA^*H9N2*^-mCor1-LysM-His-HDEL **(E)**, or CTB-His-HDEL(CTB) and GB1-CTB-His-HDEL (GB1-CTB) **(F)**. Total protein extracts from leaf tissues of *N*. *benthamiana* harvested from 3, 5, and 7 DPI were analyzed by western blotting using anti-CBM3 **(D)** or anti-His **(E,F)** antibodies. RbcL stained with CBB was used as a loading control. Lanes 1, 3, and 5 indicate target proteins without GB1 fusion, and lanes 2, 4, and 6 indicate GB1-fused target proteins.

Supplementary Figure 3 Fusion of GB1 leads to an increase in transcript levels of target genes. The indicated constructs were transiently expressed in *Nicotiana benthamiana via Agrobacterium*-mediated infiltration. Total RNA was prepared from leaf tissues at 3 dpi and used for qRT-PCR. *ACT3* was used as an internal control for qRT-PCR. **(A)** GFP and GB1-GFP. **(B)** CTB and GB1-CTB. GB1-GFP and GB1-CTB levels were represented relative to the levels of GFP and CTB, respectively. Results in panels **(A,B)** are the mean ± SE (*n* = 3). Single asterisks indicate a significant difference (Student’s *t*-test; asterisk, *P* < 0.05).

Supplementary Figure 4 Expression of GB1 mutant forms in *Nicotiana benthamiana*. Total protein extracts prepared from leaf tissues harvested at 3, 5, and 7 DPI were analyzed by western blot analysis using anti-GFP antibody. RbcL stained with CBB was used as a loading control. Lanes 1, 2, 3, and 4 indicate GFP, GB1-GFP, GB1[E27A], and GB1[E27A/W43A], respectively.

Supplementary Figure 5 Human Fc weakly interacts with GB1[W43A]. Protein extracts from *Nicotiana benthamiana* expressing the indicated constructs were used for the pull-down experiments using microcrystalline cellulose (MCC) beads. The pull-down proteins were analyzed by immunoblotting with anti-CBM3 or anti-His antibodies.
