# Supplementary figures and images for: The B1 Domain of Streptococcal Protein G Serves as a Multi-Functional Tag for Recombinant Protein Production in Plants
Source: Front Plant Sci. 2022 Apr 25;13:878677. doi: 10.3389/fpls.2022.878677 (PMC9083265; doi:10.3389/fpls.2022.878677)

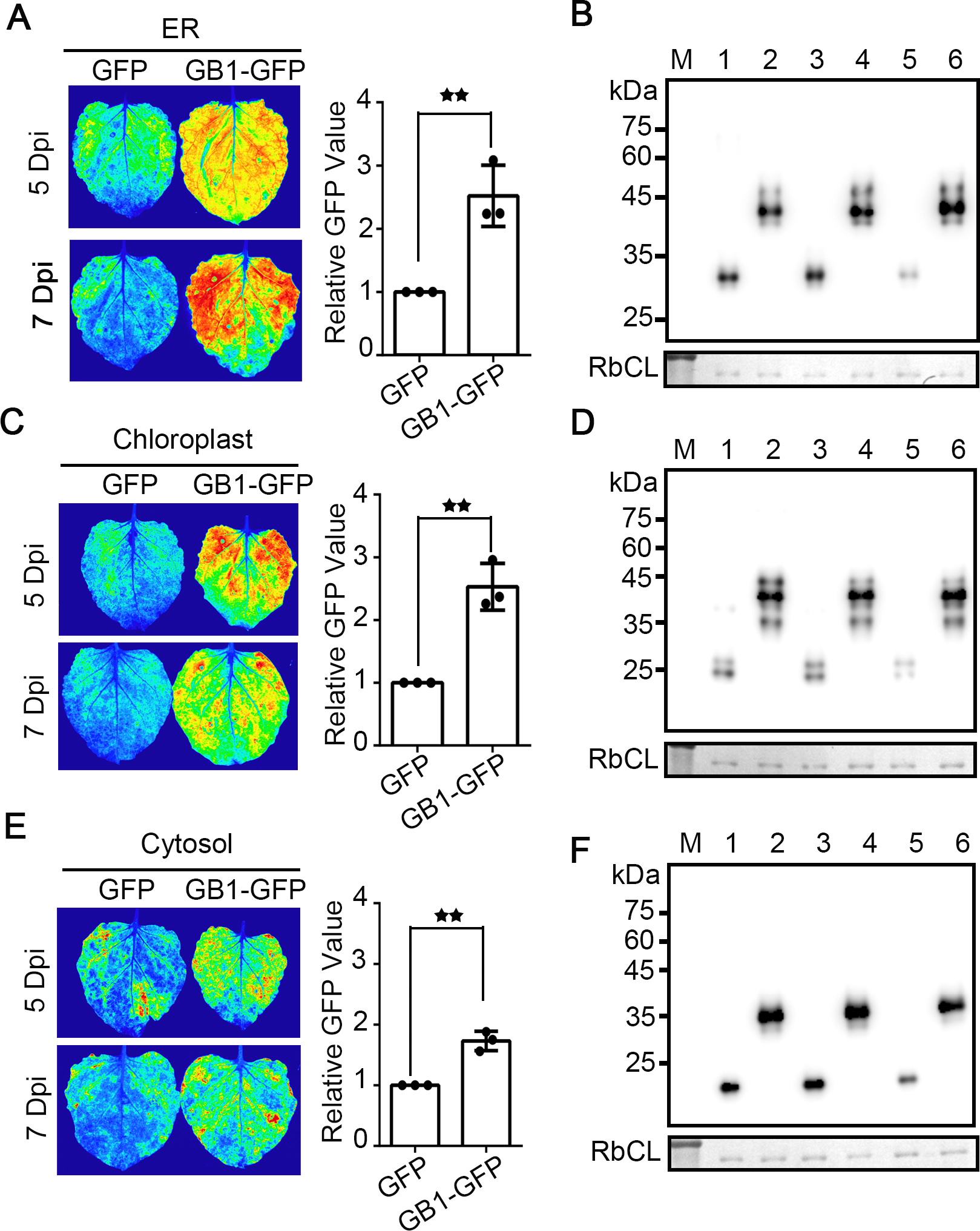

Supplement: Supplementary Figure 1 — GB1 dramatically increases the expression of soluble GFP in leaf tissues of Nicotiana benthamiana. (A,C,E) Images of GFP fluorescence for the ER (A), chloroplast (C), and cytosol (E)-localized GFP constructs. Images were taken at 5 and 7 dpi. The GFP signals were quantified at three DPI from three different samples. (B,D,F) Western blot analysis. Total protein extracts from leaf tissues harvested at 3, 5, and 7 DPI were analyzed by western blot analysis using the anti-GFP antibody. ER (B), chloroplast (D), and cytosol (F)-localized GFP and GB1-GFP at three time points. Lanes 1, 3, and 5 indicate GFP at 3, 5, and 7 DPI, respectively. RbcL stained with CBB was used as a loading control. Lanes 2, 4, and 6 indicate GB1-GFP at 3, 5, and 7 DPI, respectively. Results in panels (A,C,E) mean ± SD (n = 3). Asterisks indicate a significant difference (Student’s t-test; two asterisks, 0.001 < P < 0.05). [file Image_1.jpg]

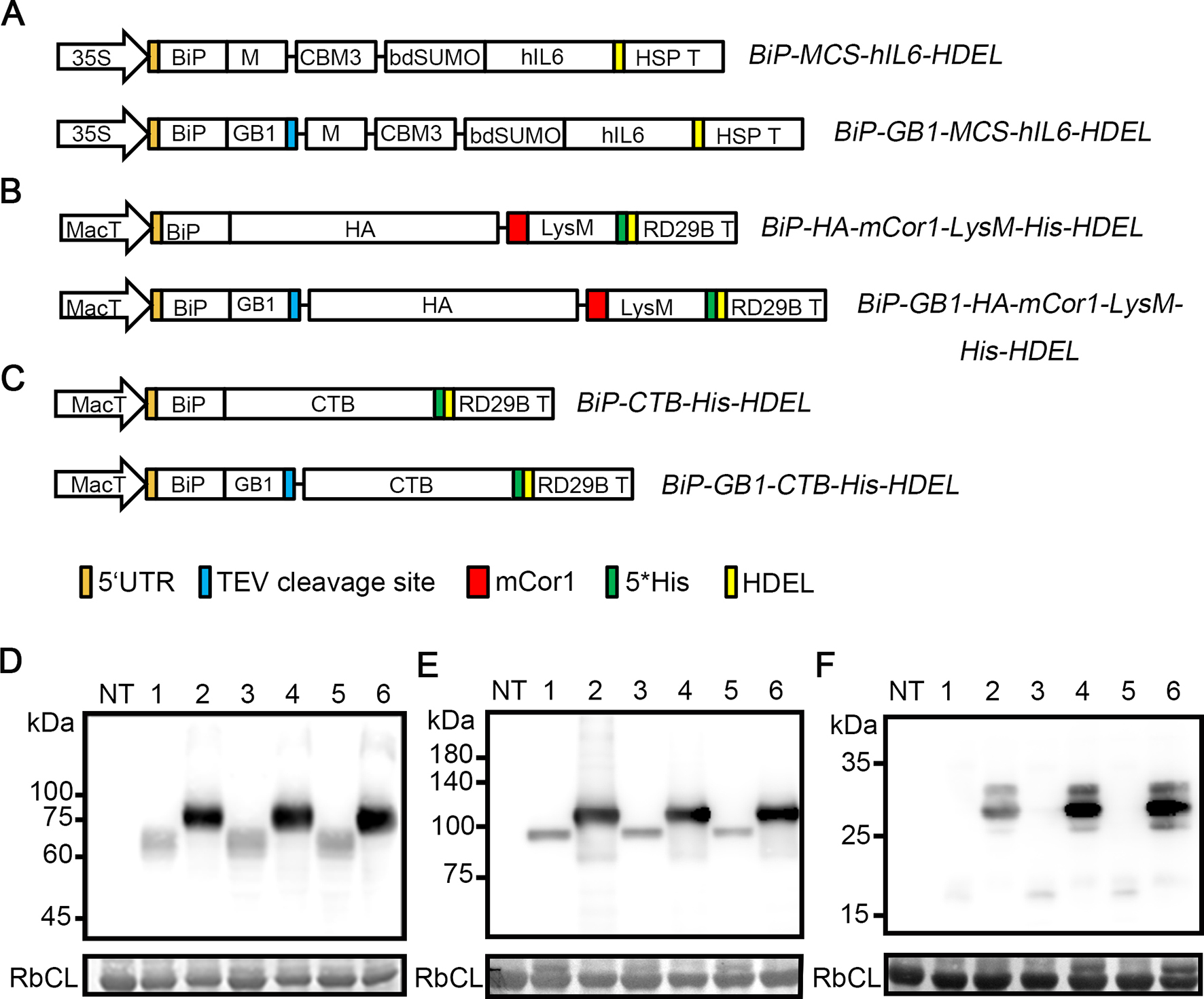

Supplement: Supplementary Figure 2 — Western blot analysis of the expression of various target genes in Nicotiana benthamiana. (A–C) Schematic representation of constructs. (D–F) Western blot analysis for the expression of various recombinant proteins. Nicotiana benthamiana leaf tissues were infiltrated with Agrobacterium harboring MCS-hIL6-HDEL(hIL6) or GB1- MCS-hIL6-HDEL(GB1-hIL6) (D), HAH9N2-mCor1-LysM-His-HDEL or GB1- HAH9N2-mCor1-LysM-His-HDEL (E), or CTB-His-HDEL(CTB) and GB1-CTB-His-HDEL (GB1-CTB) (F). Total protein extracts from leaf tissues of N. benthamiana harvested from 3, 5, and 7 DPI were analyzed by western blotting using anti-CBM3 (D) or anti-His (E,F) antibodies. RbcL stained with CBB was used as a loading control. Lanes 1, 3, and 5 indicate target proteins without GB1 fusion, and lanes 2, 4, and 6 indicate GB1-fused target proteins. [file Image_2.jpg]

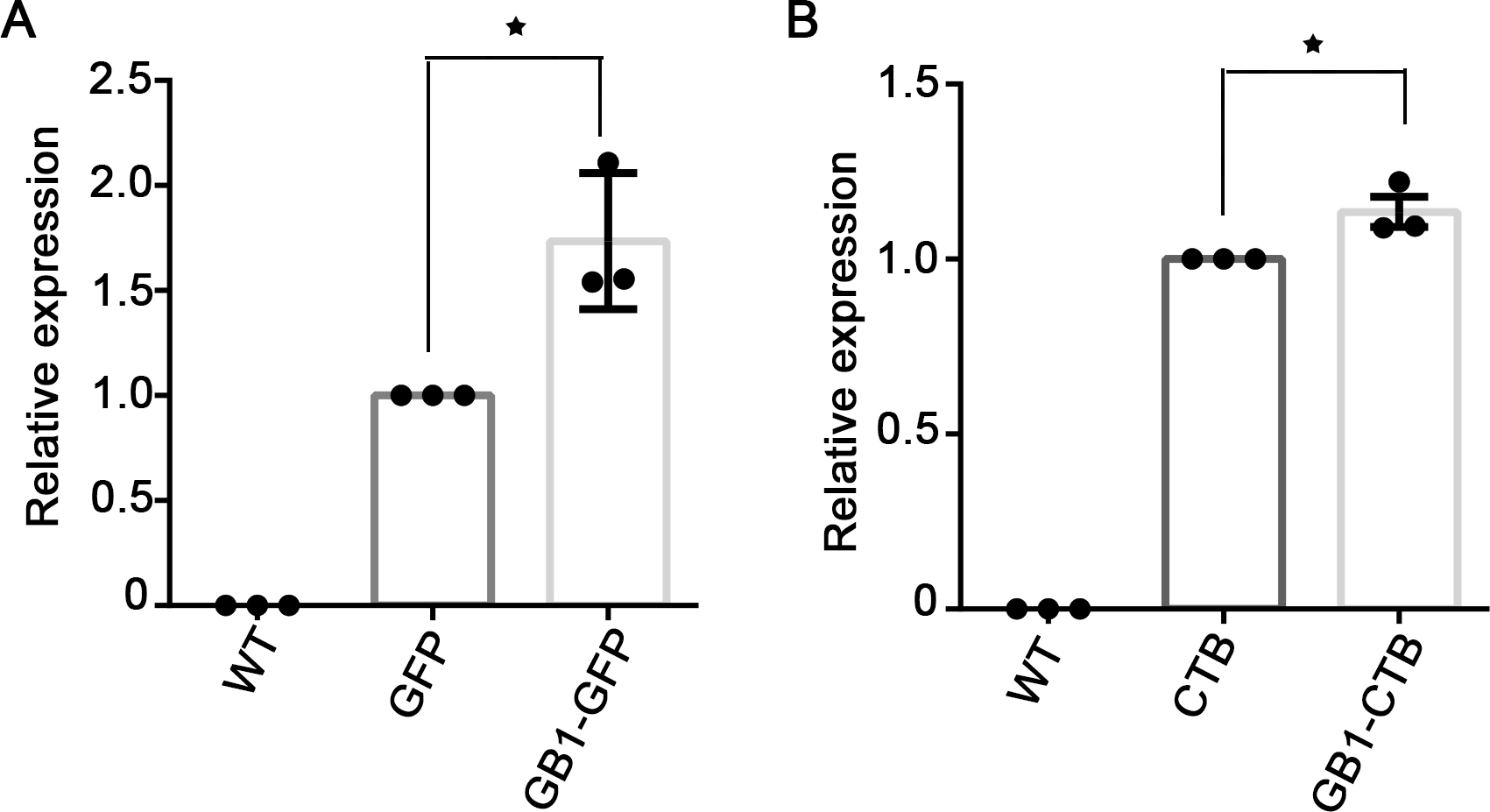

Supplement: Supplementary Figure 3 — Fusion of GB1 leads to an increase in transcript levels of target genes. The indicated constructs were transiently expressed in Nicotiana benthamiana via Agrobacterium-mediated infiltration. Total RNA was prepared from leaf tissues at 3 dpi and used for qRT-PCR. ACT3 was used as an internal control for qRT-PCR. (A) GFP and GB1-GFP. (B) CTB and GB1-CTB. GB1-GFP and GB1-CTB levels were represented relative to the levels of GFP and CTB, respectively. Results in panels (A,B) are the mean ± SE (n = 3). Single asterisks indicate a significant difference (Student’s t-test; asterisk, P < 0.05). [file Image_3.jpg]

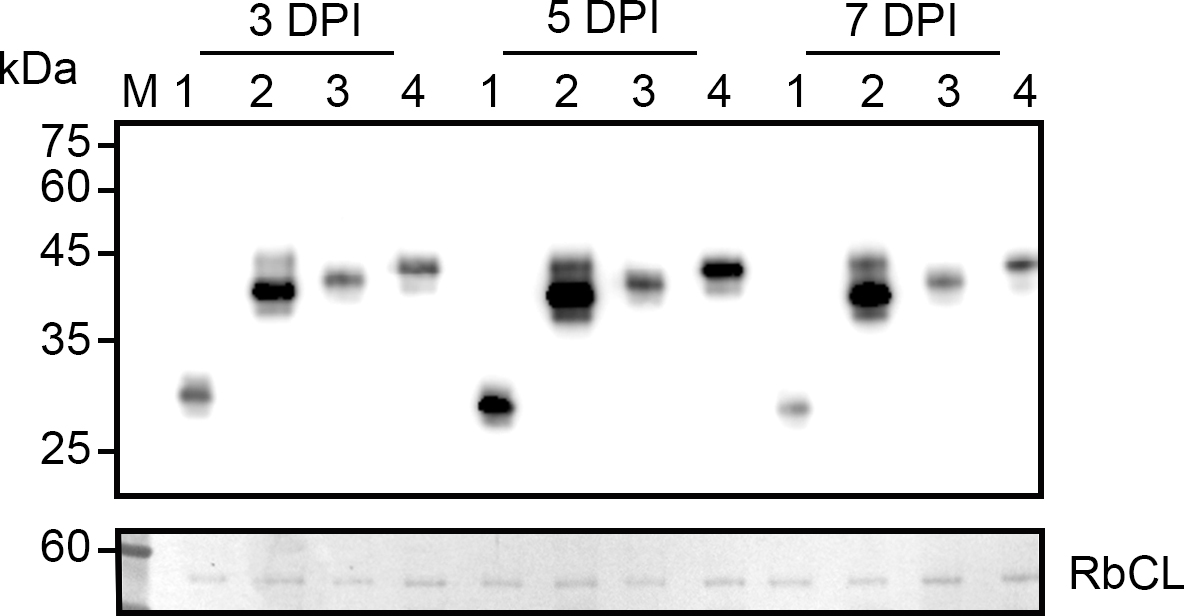

Supplement: Supplementary Figure 4 — Expression of GB1 mutant forms in Nicotiana benthamiana. Total protein extracts prepared from leaf tissues harvested at 3, 5, and 7 DPI were analyzed by western blot analysis using anti-GFP antibody. RbcL stained with CBB was used as a loading control. Lanes 1, 2, 3, and 4 indicate GFP, GB1-GFP, GB1[E27A], and GB1[E27A/W43A], respectively. [file Image_4.jpg]

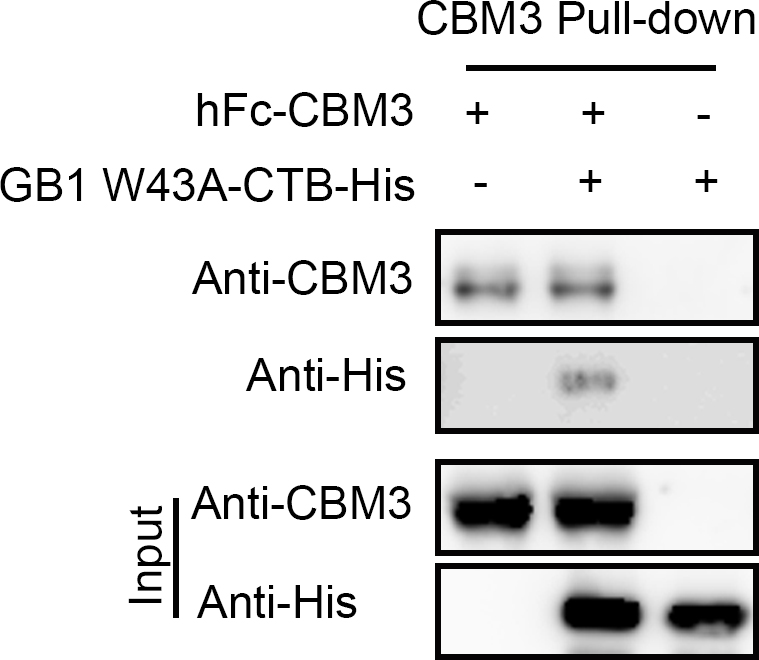

Supplement: Supplementary Figure 5 — Human Fc weakly interacts with GB1[W43A]. Protein extracts from Nicotiana benthamiana expressing the indicated constructs were used for the pull-down experiments using microcrystalline cellulose (MCC) beads. The pull-down proteins were analyzed by immunoblotting with anti-CBM3 or anti-His antibodies. [file Image_5.jpg]
