# Supplementary material for: The B1 Domain of Streptococcal Protein G Serves as a Multi-Functional Tag for Recombinant Protein Production in Plants
Source: Front Plant Sci. 2022 Apr 25;13:878677. doi: 10.3389/fpls.2022.878677 (PMC9083265; doi:10.3389/fpls.2022.878677)
Supplement: Supplementary file 6 [file Table_1.docx]

| **Supplemental Table 1. Primers used in this study.** |
| --- |
|  |

| Name | Purpose | 5’-3’ |
| --- | --- | --- |
| P1 | F-MacT-sequencing | gccttgcttcctattatatcttccc |
| P2 | R-BamHI-L-GB1 | cccGGATCCTTGAACCTCCTGAACCTCCG |
| P3 | F-GB1 W43A overlap | Cggtgtggatggtgaagcgacctacgatgatgc |
| P4 | R-GB1 W43A overlap | GCATCATCGTAGGTCGCTTCACCATCCACACCG |
| P5 | R-BamHI-RBCS | CCCGGATCCTGGAATCGGTAAGGTCAGGAAG |
| P6 | R-BamHI-L-GB1-RBCS | CCCGGATCCTTGAACCTCCTGAACCTCCTTCGGTAACAGTGAAGGTTTTG |
| P7 | F-XbaI-UTR-GFP | CCCtctagaattattacatcaaaacaaaaaatggtgagcaagggcgaggag |
| P8 | F-BamHI-AA-Luc | cgGGATCCAAatggaagacgccaaaaacataaag |
| P9 | R-PstI-Luc | gttCTGCAGttacaatttggactttccgcc |
| P10 | F-SP6 promotor | TGCCCATTCATATCCGTTCT |
| P11 | R-SV40-terminator | GTTGTTAACTTGTTTATTGCAGCT |
| qP1 | Forward, *N. benthamiana* actin, 180bp | atggaaacattgtgctcagtg |
| qP2 | Reverse, *N. benthamiana* actin, 180bP | ggtgctgagagaagccaag |
| qP3 | Forward, sGFP, 153bp, | cagcagaacacccccatc |
| qP4 | Reverse, sGFP, 153bp, | CATGCCGAGAGTGATCCC |
| qP5 | Forward, CTB, 183bp, | cctcagaacataaccgatttg |
| qP6 | Reverse, CTB, 183bp, | CTTCTGTGAATCGATATGTTGAC |
